# The Influence of Industrial Environmental Factors on Soft Robot Materials

**DOI:** 10.3390/ma16082948

**Published:** 2023-04-07

**Authors:** Dan Mihai Rusu, Olivia Laura Petrașcu, Adrian Marius Pascu, Silviu Dan Mândru

**Affiliations:** 1 Mechatronics and Machine Dynamics Department, Technical University of Cluj-Napoca, 400114 Cluj-Napoca, Romania; dan.mandru@mdm.utcluj.ro; 2 Department of Industrial Machines and Equipment, Engineering Faculty, Lucian Blaga University of Sibiu, Victoriei 10, 550024 Sibiu, Romania

**Keywords:** ecoflex silicone rubber, industrial, material behavior, soft robotics

## Abstract

This work aims to identify the effects that a series of environmental factors, specific to the industrial conditions, have on the materials in the structure of soft robots and, therefore, on soft robotics systems. The purpose is to understand the changes in the mechanical characteristics of silicone materials, with the aim of transferring soft robotics applications from the sphere of services in the industrial field. Distilled water, hydraulic oil, cooling oil, and UV rays are the environmental factors considered in which the specimens were immersed/exposed for 24 h according to ISO-62/2008. The analysis was carried out on two of the most widely used materials in the field, belonging to the category of silicone rubber, which were subjected to uniaxial tensile tests on the strength testing machine Titan 2 Universal. The results show that the greatest impact on the characteristics of the two materials was when exposed to UV rays, while the other media tested had relatively little impact on the mechanical and elastic properties (tensile strength, elongation at break, and tensile modulus) of these materials.

## 1. Introduction

The growing interest of researchers in the field of soft robotics is a fact evidenced by the increasing number of scientific publications. Several reviews highlight the evolution of the field, which, in recent years, has seen spectacular growth [1,2,3]. Latterly, applications in this field have evolved considerably, especially the characteristics of actuators, where improvements have been made in terms of actuation, modeling, manufacturing, and mechanical properties. However, for actuators to be used in real applications, analyses of their lifetime, response improvement, and the output force are needed [4]. From the literature review, soft robotics researchers are turning their attention to transitioning from soft robot applications in controlled environments and laboratory conditions to real applications where these robots can be implemented and indubitably bring benefits [5]. Based on the advantages that soft robots have over rigid body robots, they gain considerable potential in industrial applications. Advantages related to safety in human–robot or product-robot interaction, versatility, and cost will improve processes in the industrial environment and beyond [6].

To be able to integrate soft robots into industrial environments and processes, a series of analyses of their behavior is needed to validate their performance in their interaction with different aggressive agents. In industrial climates, such as manufacturing and production, they rely on equipment and processing machines that use cooling fluids and oils, hydraulic oils, and other fluids utilized in the mechanical machining process [7], including common equipment, such as industrial washing machines that use solvents and water in their degreasing process of machined parts. Another increasingly used agent in the food industry is ultraviolet rays, used as a method to eliminate micro-organisms (bacteria, viruses, yeasts, and molds), as it is non-chemical and environmentally friendly [8]. Due to the diversity of existing industries and the specific characteristics of each, there is a wide range of agents with which soft robots interact, and for which analyses of the materials’ behavior are required. Therefore, we have focused strictly on the interaction of Ecoflex 00-10 and 00-30 materials in the construction of soft robots with fluids and agents often encountered in industrial environments of mechanical processing and the food industry.

The two mentioned materials (Smooth-on, Inc., Macungie, PA, USA) were not chosen by chance for the present analysis. These two are among the most widely used in the field, with a wide range of applications, such as biologically inspired [9,10] and medical applications [11,12]. In the category of soft robotics, the most used silicone materials by researchers are Ecoflex 00-10 to 00-50, Dragon Skin, Smooth-Sil, and Elastosil M4601 [3,13], as these have different mechanical characteristics. In the literature found on the Ecoflex 00-10 to 00-50 range of silicone rubbers, some analyses were identified that dealt with their mechanical behavior under the influence of different factors.

In the article by Jennifer C. Case et al. [14], the Ecoflex 00-30 material along with two other representative materials used in the construction of soft robots were analyzed regarding the mechanical behavior through uniaxial tensile tests, cyclic loading tests, and stress relaxation tests in order to facilitate their dynamic modeling. The results show that all the materials analyzed have time-dependent nonlinear characteristics exhibiting the Mullins effect, where there is a variation of material properties in the first cycle compared to the rest. Luc Marechal et al. [15,16] provide researchers with an open-access constitutive model database called “Soft Robotics Materials Database” of the most used materials in soft robotics. Their aim being to help researchers in obtaining relevant data in the simulation process using finite element methods of soft robotic structures.. The 17 elastomeric materials analyzed were subjected to uniaxial tensile tests based on ASTM D412. Contingent to the test results on the Instron 5569 machine, parameters for hyper-elastic material models were derived and determined by nonlinear methods. Zisheng Liao et al. [17,18] addressed the thermomechanical behavior of Ecoflex 00-30 in their paper to determine the influence of temperature ranging from −40 °C to 140 °C performed on an Instron 5567 tensile testing machine with climatic chamber. The tests performed under the influence of temperature were load-unload cycles, the Mullins effect tests at different stress rates and strains, single-stage relaxing tests, and others. The results show that the temperature sensitivity of Ecoflex 00-30 differs more or less with temperature variations. Additionally, this material undergoes softening stress in the first few cycles, considering that these softenings gradually recover over time. The same authors continued the study on four Ecoflex materials with different Shore hardness (00-50, 00-30, 00-20, 00-10), performing uniaxial, echibiaxial, and plane fatigue tests on the material to determine the stress recovery behavior after the first test cycle. The results show that with the strain level increasing, the stress softening increases, and this implicitly leads to a slower stress recovery related to softening dissipation. In a microscopic analysis, the strain softening is dependent on the microscopic deformation of the polymer chains, resulting in higher strain leading to higher breakage of the rubber matrix and fillers, requiring more energy and a longer time to restore the polymeric bonds of the material.

The main problem identified is the lack of present research concerning relevant data on the behavior of the main materials used in the construction of soft robots under the influence of industrial environmental factors, such as fluids and UV radiation. Therefore, this paper aims to determine the influence of the mentioned media on two materials with different stiffnesses (Ecoflex 00-10 and 00-30), through experimental uniaxial tensile tests to failure, which can provide sufficient characteristics needed to determine the impact that the respective environment had on the material.

This study aims to provide soft robotics researchers with relevant data on the behavior of Ecoflex 00-10 and 00-30 used in the construction of soft robots under the influence of industrial environmental factors in order to help their introduction into real applications (industrial or commercial). A clear methodology for carrying out the necessary test steps based on standards that are unanimously accepted by the scientific community has been provided.

## 2. Materials and Methods

The two elastomeric materials used in this study are Ecoflex 00-10 and Ecoflex 00-30, produced by Smooth-On in the USA. These materials have different characteristics, such as shore hardness, both are transparent in color and consist of two components: part A (base) and part B (catalyst). These two parts are mixed in equal quantities and left to harden for about 4 h at room temperature. The material properties provided by the manufacturer are centralized in Table A1 in the Appendix A [19,20].

**Figure 1:**
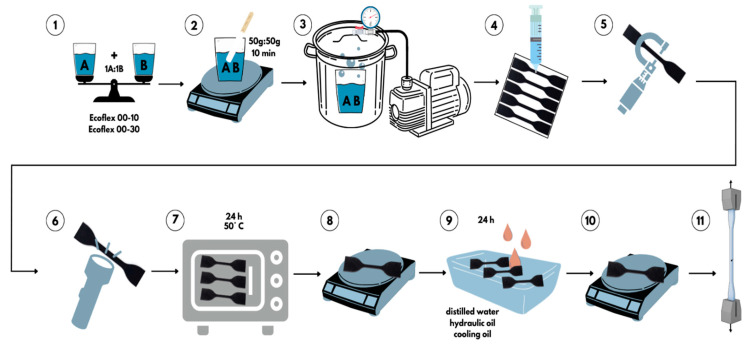
Specimen casting stages: 1—Part A and Part B in equal quantities, 2—Mixing the two parts, 3—Removal of air bubbles with the vacuuming system, 4—Pouring into the mold and left to harden for 4 h, 5—Measurement of specimens, 6—Qualitative inspection of specimens, 7—Drying specimens in the oven, 8—Weighing of specimens after drying, 9—Submerge specimens in fluid for 24 h, 10—Measuring the absorption level, 11—Uniaxial tensile tests on Titan 2 Universal machine.

Due to the large variety of experimental methods used in performing uniaxial tensile tests of elastomeric materials in the domain of soft robotics and to help establish clear working methods in this field, the methods proposed and used in the article by Luc Marechal et al. [16] were adopted as a present working methodology. For the realization of the specimens, we adopted the ASTM D412 standard (test method A) corresponding to elastomeric and vulcanized rubber materials [21]. The specimen template dimensions are 115 mm long, 3 mm thick, and 25 mm wide, according to the above-mentioned standard “Die C” specimen [22]. A 3D-printer-Ultimaker 3 was used to prepare the negative molding material, the mold was made of ABS (acrylonitrile butadiene styrene) with a nozzle diameter of 0.4 mm. To shorten the time required to make the test specimens, a set of five negative frames, to pour the rubber in liquid form, was made. The working steps for making the test specimens are shown in Figure 1. The two components were weighed with a precision scale in the amount of 50 g—Part A and 50 g—Part B (step 1), and mixed for 10 min to homogenize well (step 2). The resulting mixture was introduced into the vacuuming machine to remove air bubbles from the material structure (step 3). This step is very important in the quality of the tests performed because the bubbles can lead to changes in the mechanical characteristics. Afterward, the mixture was molded in the frame manufactured on the 3D printer, and a set of five specimens was obtained in a single molding step, which resulted in a shortening of the manufacturing time (step 4). The material was left to cast for 4 h, then removed from the mold. In steps 5 and 6, a qualitative inspection of the specimens was carried out, they were measured and visually inspected for dimensional corresponding and air bubbles in the specimen structure. Steps 7–10 are part of the ISO-62/2008 standard on the determination of water absorption in plastics. This international standard provides a clear procedure for water absorption in specimens.

The standard requires the use of distilled water in which specimens are immersed and held for 24 h. The methodology of the standard requires a set of steps, namely the specimens are first oven-dried for 24 h at 50 °C, according to step 7, in the next step they are weighed using a precision scale (step 8). In step 9, the specimens are immersed in a glass dish in order not to influence the quality of the tests and left for 24 h. The media in which the specimens were immersed are distilled water, synthetic hydraulic oil (Divinol HLP ISO 32, viscosity: 32 mm²/s (40 °C)), and synthetic cooling oil (AZUR-CUT 602.01 M-15, viscosity: 15 mm²/s (40 °C)). These two synthetics are a combination of mineral oils with additives that are specifically designed to operate at high pressures and cope with highly intense machining regimes by material removal. This standard applies only to the three liquid media (distilled water, hydraulic oil, and cooling oil), the rest of the media in the analysis (ambient environment and UV radiation) did not use this standard. Tests under the influence of UV radiation were carried out using a 38 W UV lamp. The specimens’ entire surface was exposed at a distance of approximately 50 mm from the lamp. After 24 h, they were wiped with an absorbent towel and dried thoroughly. In step 10, after drying, the specimens were again weighed to determine the amount of liquid absorbed. To calculate the liquid’s absorption, Formula (1) was used:

(1) $$c=\frac{m_{2}-m_{1}}{m_{1}}\cdot 100\%$$

where *c* represents the percentage by mass of liquid absorbed, the mass of the test specimen after initial drying before immersion is noted with *m*_1_, and *m*_2_ expresses the mass after immersion.

**Figure 2:**
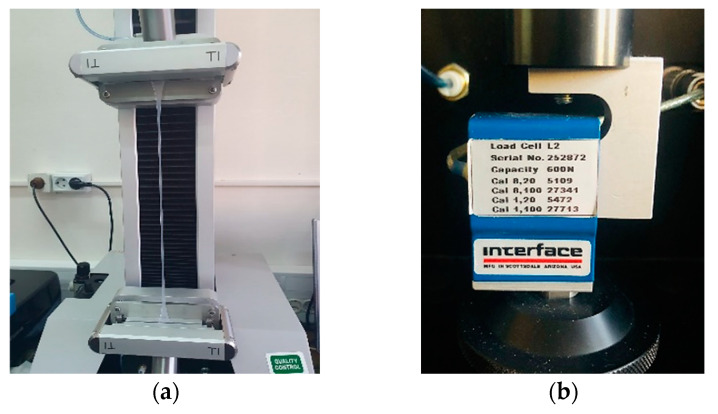
TITAN 2 tensile testing machine, (**a**) Catching specimens in machine jaws, (**b**) 600 N force cell.

In the final stage, specimens were tested at an ambient temperature of 21 °C in uniaxial tensile on the Titan 2 Universal tensile testing machine, presented in Figure 2a, until breakage. Data acquisition was performed using the dedicated machine strength tester software (software version 7.0.4.14642). The testing speed of the tests equals 50 mm/min and the testing machine was equipped with a 600 N force cell, visible in Figure 2b. Particular attention was paid to the specimen gripping in the tensile test machine’s jaws so that this area of the machine’s jaws covered as much as possible of the dumbbell sample specimen area for high efficiency. The gripping jaws have a pneumatic actuator and provide an adherent elastomeric material on their surface that maintains a good grip in the machine jaws, preventing slippage during testing.

## 3. Results

The results obtained from the tests, in terms of the level of absorption and the uniaxial tensile tests performed, are presented structured in two parts, one for each material. For the quality of the tests, it was decided to select only the five most representative tests from the data sets obtained. Additionally, from these five selected representative data sets, the results were presented as their mean value. Both types of materials were subjected to uniaxial tensile tests, mentioning that outliers were removed from the initial sets of tests, retaining the five representative tests for each material.

The maximum tensile stress, tensile strain, maximum true stress, maximum true strain, maximum tensile modulus, and tensile modulus (E), for each of the twenty-five specimens, are shown in Table 1. The maximum of each of the above-mentioned sizes was calculated in Excel after extracting the data from the machine after testing. Table 1 also shows the average values for each material tested.

Subsequently, the normal distribution of the experimentally obtained data for each set of specimens was checked using the statistical functions provided in Excel. In this statistical analysis, the mean values of the experimentally determined quantities (stress, strain, and tensile modulus) were calculated, and the standard mean square deviation and standard variation. The minimal statistical analysis shows that the tests’ results have a distribution close to the normal distribution and that there are no outliers in the experimentally obtained value strings, which leads to the conclusion that the tests were correctly performed, and therefore results are accurate as well.

Table 1 also contains the values of true stress σt and true strain εt calculated with the following formulas, where σe is engineering stress and εe represents engineering strain:

(2) $$\sigma_{t}=\sigma_{e}\cdot \left(1+\varepsilon_{e}\right)\;\left[\mathrm{MPa}\right]$$

(3) $$\varepsilon_{t\;}=\ln\left(1+\varepsilon_{e\;}\right)\left[\mathrm{mm}/\mathrm{mm}\right]$$

All experimental data obtained after the test running was followed by a statistical analysis, where we calculated the average value, standard mean square deviation (4), and the standard variation (5), with the calculation relations:

(4) $$\;\sigma =\sqrt{\frac{\sum_{i=1}^{n}{\left(x_{i\;}-m\right)}^{2}}{n}}$$

(5) $$S=\sqrt{\frac{\sum_{i=1}^{n}{\left(x_{i\;}-\bar{x}\right)}^{2}}{n-1}}\;$$

### 3.1. Results Obtained for Ecoflex 00-30

We performed tensile tests on 00-30 specimens that have been maintained in different environments to determine the influence of these on the mechanical and elastic characteristics. All tests were performed with a strain rate of 0.02 s^−1^.

**Figure 3:**
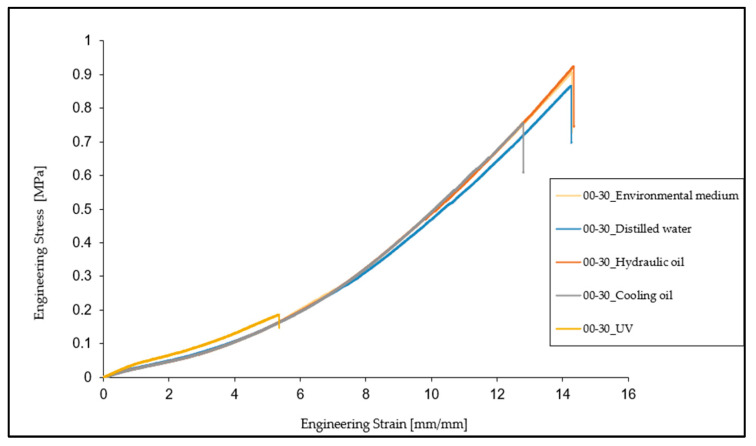
Engineering stress vs. engineering strain curves for Ecoflex 00-30 in the five different environments.

Based on the experimental results, a characteristic engineering stress vs. engineering strain curve was plotted for each of the five sets of tests for the particular environments, as shown in Figure 3.

From the comparison of the results of these tests, it can be observed that the mechanical and elastic properties of the material (tensile strength, elongation at break, and tensile modulus) did not undergo major changes regarding specimens kept in distilled water and hydraulic oil. In the case of cooling oil, a decrease of 16.43% in the breaking strength and 10.20% in the elongation at break was observed. As regards tensile modulus, it remains very close to the value determined for specimens tested under normal conditions, unaffected by interaction with any aggressive medium.

In the case of specimens subjected to ultraviolet (UV) radiation, there is a significant decrease in the breaking strength and elongation at the break by 78.12% and 59.90%, respectively, compared to normal conditions specimens. In terms of tensile modulus, this time there is an increase of approximately 37.07% compared to the initial state of the specimens.

Concerning how the absorption level of a liquid in the tested material influences its mechanical and elastic characteristics, the absorption level of the three liquid media are presented in Table 2, Table 3 and Table 4. The absorption percentage was calculated with Formula (1).

Table 2, Table 3 and Table 4 show the numerical values of absorbance expressed in grams, before and after immersion in the liquid medium for 24 h, of each of the five specimens.

**Figure 4:**
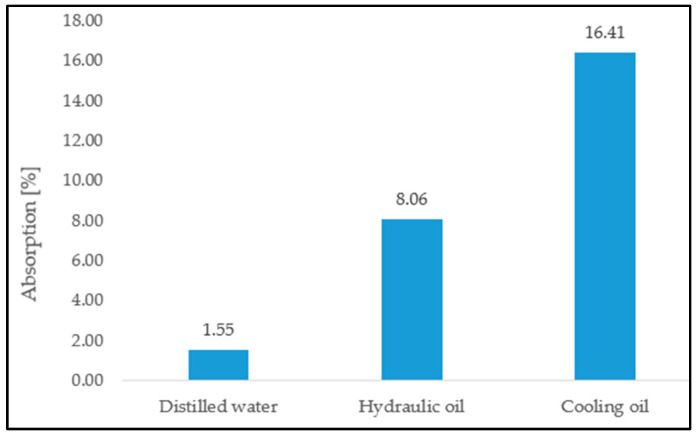
Average absorption level in Ecoflex 00-30 specimens in the three liquid environments.

From Figure 4, it can be seen that the most significant absorption is for cooling oil, followed by hydraulic oil and distilled water. The absorption of cooling oil compared to hydraulic oil is about twice as high, and compared to distilled water, about 10 times higher.

**Figure 5:**
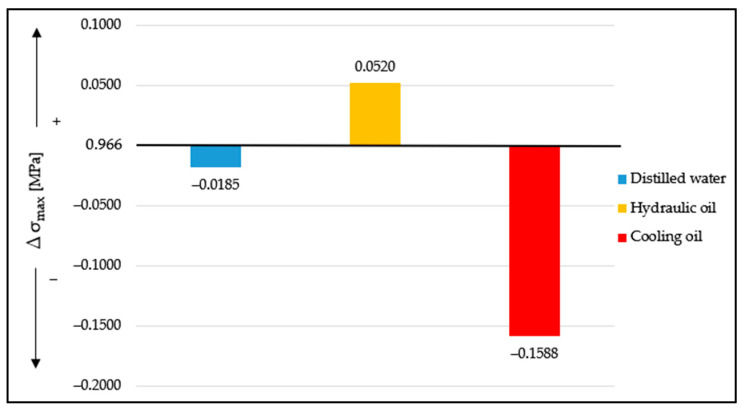
Comparison of the average of maximum values of tensile stress for Ecoflex 00-30 specimens for each liquid medium.

Figure 5 shows the comparisons of the maximum values of tensile stress for the cases of the three liquid media relative to the averages of the specimens tested in the ambient medium. The origin of the graph or starting point of the increase and decrease in the values of the tensile stresses in the specimen is 0.9660 MPa, representing the maximum value for the specimens tested in an ambient medium, as shown in Figure 5.

It can be observed that, in the case of distilled water, at an absorption of 1.55%, the maximum tensile stress at which the specimen breaks decreases by the absolute value of 0.0185, referring to the values of stresses and deformations related to the ambient medium. In the case of hydraulic oil, at an absorption of 8.06%, the maximum tensile stress at which the specimen breaks, increases by the absolute value of 0.052, and in the case of cooling oil, at an absorption of 16.41%, the maximum value of the tensile stress shows a decrease by the absolute value of 0.1588.

**Figure 6:**
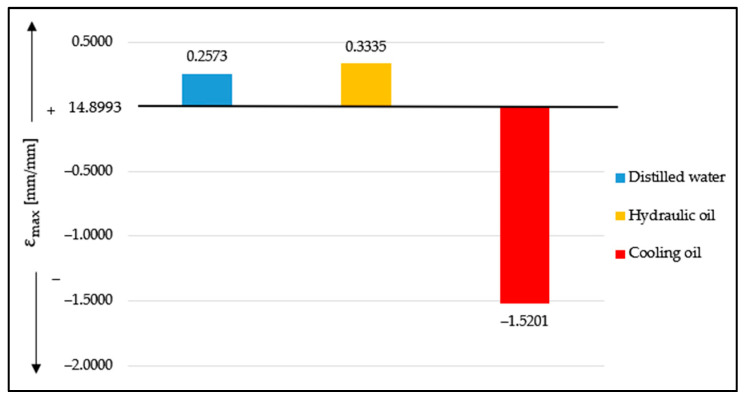
Comparison of the average maximum values of tensile stress for Ecoflex 00-10 specimens for each liquid medium.

Figure 6 shows the comparisons of the maximum values of tensile strain for the cases of the three liquid media relatives to the averages of the specimens tested in the ambient medium. The origin of the graph is 14.8993 mm/mm.

It can be observed that in the case of distilled water, at an absorption of 1.55%, the maximum tensile strain, at which the specimen breaks, increases by the absolute value of 2.257. In the case of hydraulic oil, at an absorption of 8.06%, the maximum tensile strain increases by the absolute value of 0.3335, and, in the case of cooling oil, at an absorption of 16.41%, the maximum value of the tensile strain shows a decrease of 1.5201.

It can be concluded that there is not necessarily a correlation between the level or amount of liquid absorbed by the specimen/material and the increase/decrease in tensile stress/tensile strain, but rather the influence of chemical factors in the external liquid environment on the mechanical and elastic properties of the material.

### 3.2. Results Obtained for Ecoflex 00-10

We also performed tensile tests on specimens made of Ecoflex 00-10 that have been maintained in different environments to determine their influence on its mechanical and elastic characteristics. Thus, we put the specimens in distilled water, hydraulic oil, cooling oil, and under UV radiation. All tests were performed with a strain rate of 0.02 s^−1^ and outliers were removed from the initial sets of tests, retaining the five representatives.

The maximum tensile stress, tensile strain, maximum true stress, maximum true strain, and maximum tensile modulus for each of the twenty-five specimens are shown in Table 5. This table also shows the average values for each material tested.

The values for true stress and true strain were calculated with Formulas (2) and (3). We also performed a statistical analysis consisting of the standard mean square deviation and the standard variation with Formulas (4) and (5), as in the case of specimens 00-30.

For the Ecoflex 00-10 material, unfortunately, we were unable to determine the maximum tensile stress and maximum tensile strain due to the limitations imposed by the testing machine, which has too short a stroke for the elongation of this material. Thus, we could only make a comparison of the tensile modulus which was calculated up to the tensile strain value of 0.5 mm/mm.

**Figure 7:**
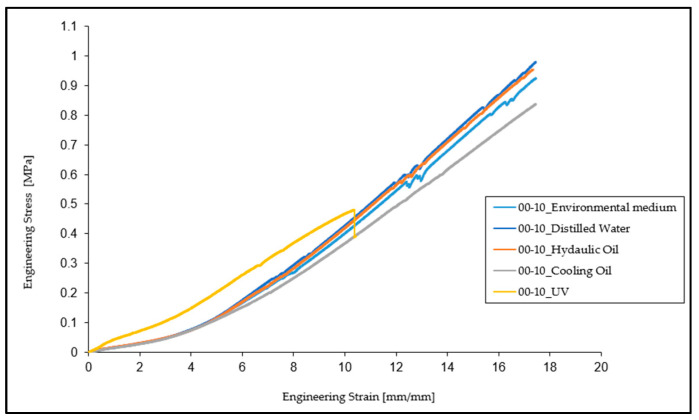
Average of the five specimens tested for Ecoflex 00-10 in the five environments.

We can observe, however, that the specimens subjected to UV reached breakage due to degradation of the material and also a significant decrease in the breaking strength and elongation at break compared to the specimens tested under normal conditions. As regards the tensile modulus for the specimens subjected to UV, an increase of 125.66% compared to the normal conditions is observed, as shown in Figure 7.

To determine how the absorption level of a liquid in the tested material influences the mechanical and elastic characteristics of the material, the absorption level of the three liquid media in Ecoflex 00-10 was determined and presented in Table 6, Table 7 and Table 8. The absorption percentage was calculated with Formula (1). In this case, as in the case of Ecoflex 00-30, cooling oil has a significant impact on liquid absorption compared to the other media analyzed. The absorption ratio between the three media remains approximately the same.

In the case of specimens made of Ecoflex 00-10 material, we could not make a comparison of the maximum tensile stress and tensile strain values because these specimens did not reach the breaking point due to the limitations imposed by the uniaxial testing machine.

### 3.3. Comparative Analysis of Specimens from Each Environment

**Figure 8:**
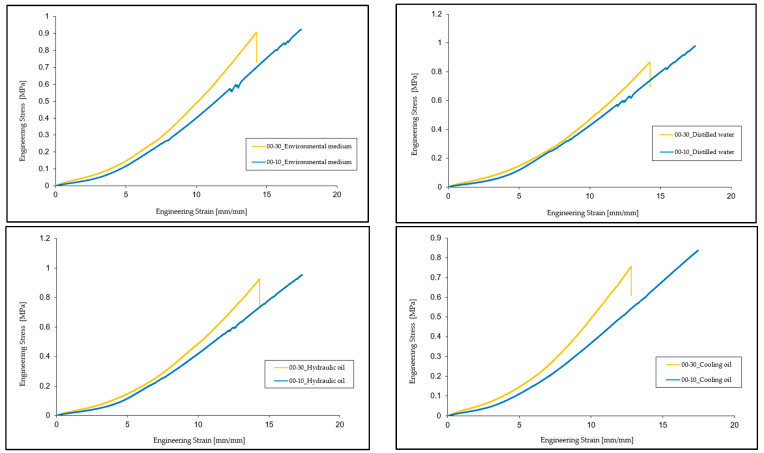
Comparative average of specimens 00-30 and 00-10 in ambient medium, distilled water, hydraulic oil, and cooling oil.

When comparing the test specimen media tested in the ambient medium, distilled water, hydraulic oil, and cooling oil, it can be seen that the Ecoflex 00-30 specimens reached the breaking point, while the Ecoflex 00-10 specimens did not break due to the too short stroke of the uniaxial test machine. Thus, we compared the modulus of elasticity of the two types of materials. In the case of Ecoflex 00-10, tested in an ambient environment, a decrease in the modulus of elasticity compared to Ecoflex 00-30 test specimens of about 45% is observed. Regarding distilled water, the test of 00-10 specimens shows a decrease in the modulus of elasticity compared to 00-30 test specimens by approximately 40.66%. As for hydraulic oil, the modulus of elasticity of Ecoflex 00-10 test specimens decreases by approximately 35.08% compared to that of 00-30 test specimens, and in the case of cooling oil, the modulus of elasticity of 00-10 test specimens decreases by approximately 41.27% compared to 00-30. It is also observed that the characteristic curves of the materials are similar up to the point where deviations occur, in the 00-10 material, as shown in Figure 8.

**Figure 9:**
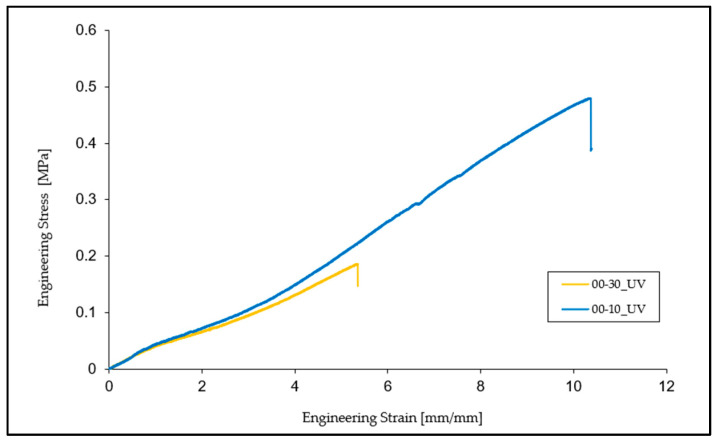
Comparative average of specimens 00-30 and 00-10 at UV.

Comparing the test specimen tested under UV, it can be seen that both types of material have reached the breaking point. The comparison shows that the mechanical and elastic properties of the material (tensile strength, elongation at break, and tensile modulus) underwent major changes after being exposed to UV radiation for 24 h. Observing 00-10, there is an increase in the breaking strength by 142.73% and an increase in the elongation at break by 93.73%, while the tensile modulus remains very close to that of 00-30, with a decrease of approximately 4.1%, as shown in Figure 9.

## 4. Discussion and Conclusions

This work addressed the impact of fluidic environments, specific to the industry, on the mechanical and elastic characteristics of elastomeric materials (Ecoflex 00-10 and 00-30) belonging to the soft robotics field. The purpose is to help transfer the field of soft robotics from the service sphere to that of industrial applications. Based on the international standard ISO-62/2008, impact uniaxial tensile tests until the specimens broke according to ASTM D412 standards were carried out on the two materials, using five common fluidic media: hydraulic oil, cooling oil, distilled water, and UV rays.

Within the results section, several issues require further detail. The first aspect concerns the absorption of the two materials. Observing the results cooling oil has the highest absorption rate, followed by hydraulic oil, then distilled water, with this ratio being maintained for both materials. Related to this, there may be a few factors that can influence the absorption rate, but in this case, on the one hand, the determining factor is related to the chemical composition of the liquid medium in which the specimens were immersed. On the other hand, a few reasons can be attributed to the absorption level, such as the different viscosity levels of the liquid media, but this is not validated in this work since viscosity did not influence the absorption level.

Another aspect that needs to be detailed is related to the occurrence of deviations from the characteristic curve in the case of Ecoflex 00-10. These deviations with different amplitudes depending on the test medium occur only in the case of 00-10, which would exclude several factors, such as the settings of the tensile testing machine. From the comparative analysis in Figure 8 and Figure 9, it can be seen that the deviations with the largest oscillation occur in the case of the specimens tested in the ambient environment, while in the case of the other environments, there is a certain uniformity. Additionally, it needs to be pointed out that the position of the deviations in the five environments is somewhat identified at similar stress and strain levels, and also the occurrence interval is approximately equal.

Noticeably, in the case of the five environments, these oscillations appear on the characteristic curve at certain intervals, in the case of the ambient medium, the first deviation appears at a tensile strain of 7.88 mm/mm and tensile stress of 0.26 MPa, followed by a second, more evident deviation, at 12.68 mm/mm and 0.58 MPa, and the third and last deviation appears at the interval 16.33 mm/mm and 0.83 MPa.

In the case of distilled water, the characteristic curve also shows deviations, but less obvious, at 7.57 mm/mm and 0.26 MPa, at 12.47 mm/mm and 0.59 MPa, and at 16.10 mm/mm and 0.88 MPa, being close to the values at which these deviations appeared in the case of the initial medium. As for hydraulic oil, these deviations appear on the characteristic curve but are very little obvious, namely at 7.15 mm/mm strain and 0.23 MPa stress, at 12.81 mm/mm and 0.61 MPa, and 15.08 mm/mm and 0.79 MPa. The last case is that of cooling oil, where these deviations are not obvious, the curve being linear, while in the case of UV, the deviation appears at 6.73 mm/mm and 0.29 MPa.

**Figure 10:**
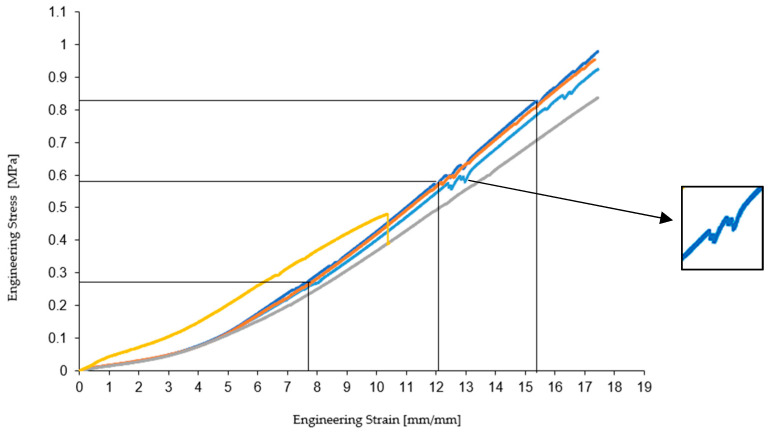
Deviations on characteristic curve.

There is a cyclicity for the occurrence of these deviations on the characteristic curves of about 4.27 mm/mm in terms of tensile strain and 0.29 MPa in terms of tensile stress. Figure 10 shows deflections on the characteristic curves after the uniaxial tensile test.

For Ecoflex 00-30, the highest value of tensile stress (0.92 MPa) and of tensile strain (14.44 mm/mm) occurs for specimens maintained in hydraulic oil. As for the Ecoflex 00-10, we cannot accurately specify the maximum values of stress and strain, since the specimens did not reach the breaking point because of the uniaxial testing machine stroke.

As far as future directions are concerned, further analysis of the Ecoflex 00-10 is needed, both in terms of determining the material’s fracture toughness and the impact that the media analyzed in this work have on the structural and microscopic level of the material in particular. We want future analyses to focus on the determination of the behavior of other materials intensively used in soft robotics and the use of different relevant fluidic media depending on the specific application for which the soft robot was made.

Moreover, another future direction we are considering is related to the development of a soft robotics system with industrial applicability and verification of the influence of environmental factors on it.

## Figures and Tables

**Table 1:** Dimensions of 00-30 specimens, maximum tensile stress, maximum tensile strain, maximum true stress, maximum true strain, and maximum tensile modulus.

Specimen No.	Specimen Dimensions		σ_max_ [MPa]	ε_max_ [mm/ mm]	σ_true max_ [MPa]	ε_true max_ [mm/ mm]	E [MPa]
	b [mm]	t [mm]					
Ambient medium							
#1	6	3	0.8815	14.2890	13.4767	2.7271	0.0340
#2	6	3	0.8897	14.3728	13.6774	2.7326	0.0312
#3	6	3	1.0082	15.4117	16.5456	2.7980	0.0321
#4	6	3	1.0097	14.9656	16.1197	2.7704	0.0318
#5	6	3	1.0411	15.4572	17.1342	2.8008	0.0316
Average value			0.9660	14.8993	15.3907	2.7658	0.0321
Standard mean square deviation			0.0747	0.5540	1.6959	0.0349	0.0011
Standard variation			0.0056	0.3069	2.8760	0.0012	0.0000
Distilled water							
#1	6	3	0.9494	15.0695	15.1529	2.7769	0.0322
#2	6	3	0.9950	15.5171	16.3277	2.8044	0.0319
#3	6	3	1.0167	15.6586	16.8377	2.8129	0.0360
#4	6	3	0.9264	15.1869	14.9087	2.7842	0.0332
#5	6	3	0.8498	14.3508	12.9561	2.7312	0.0325
Average value			0.9475	15.1566	15.2366	2.7819	0.0332
Standard mean square deviation			0.0652	0.5099	1.5058	0.0319	0.0016
Standard variation			0.0043	0.2599	2.2673	0.0010	0.0000
Hydraulic oil							
#1	6	3	0.9465	15.2875	15.3581	2.7904	0.0296
#2	6	3	0.8950	14.4409	13.7201	2.7370	0.0278
#3	6	3	1.0671	15.3654	17.4015	2.7952	0.0279
#4	6	3	1.0545	15.1185	16.9262	2.7800	0.0324
#5	6	3	1.1290	15.9519	19.0438	2.8304	0.0349
Average value			1.0184	15.2328	16.4899	2.7866	0.0305
Standard mean square deviation			0.0953	0.5427	2.0308	0.0336	0.0031
Standard variation			0.0091	0.2945	4.1241	0.0011	0.0000
Cooling oil							
#1	6	3	0.8608	13.4228	12.3572	2.6688	0.0333
#2	6	3	0.8824	13.8341	13.0635	2.6969	0.0305
#3	6	3	0.7708	13.4346	11.0919	2.6696	0.0290
#4	6	3	0.7374	12.8812	10.1708	2.6305	0.0288
#5	6	3	0.7845	13.3234	11.0610	2.6619	0.0275
Average value			0.8072	13.3792	11.5489	2.6656	0.0298
Standard mean square deviation			0.8072	13.3792	11.5489	2.6656	0.0298
Standard variation			0.0617	0.3402	1.1504	0.0237	0.0022
UV							
#1	6	3	0.1889	5.4092	1.1948	1.8577	0.0394
#2	6	3	0.1790	5.6898	1.1824	1.9006	0.0577
#3	6	3	0.2661	6.5702	1.9961	2.0242	0.0349
#4	6	3	0.2312	6.5865	1.7370	2.0264	0.0366
#5	6	3	0.1913	5.6135	1.2516	1.8891	0.0516
Average value			0.2113	5.9738	1.4724	1.9396	0.0440
Standard mean square deviation			0.0366	0.5613	0.3722	0.0798	0.0100
Standard variation			0.0013	0.3151	0.1385	0.0064	0.0001

**Table 2:** Distilled water absorption of Ecoflex 00-30 test specimens.

No. Specimen	Mass after Drying [g]	Mass after 24 h in Distilled Water [g]	Absorption [%]
#1	6.00	6.16	2.67
#2	5.58	5.66	1.43
#3	5.90	6.00	1.69
#4	5.55	5.56	0.18
#5	5.56	5.66	1.80
Average value			1.55

**Table 3:** Hydraulic oil absorption of Ecoflex 00-30 test specimens.

No. Specimen	Mass after Drying [g]	Mass after 24 h in Distilled Water [g]	Absorption [%]
#1	5.52	5.85	5.98
#2	5.28	5.75	8.90
#3	5.61	6.43	14.62
#4	5.48	6.00	9.49
#5	6.00	6.08	1.33
Average value			8.06

**Table 4:** Cooling oil absorption of Ecoflex 00-30 test specimens.

No. Specimen	Mass after Drying [g]	Mass after 24 h in Distilled Water [g]	Absorption [%]
#1	6.00	6.80	13.33
#2	5.81	6.53	12.39
#3	5.49	6.25	13.84
#4	5.76	6.65	15.45
#5	5.40	6.86	27.04
Average value			16.41

**Table 5:** Dimensions of 00-10 specimens, maximum tensile stress, maximum tensile strain, maximum true stress, maximum true strain, and maximum tensile modulus.

Scheme	Specimen Dimensions		σ_max_ [MPa]	ε_max_ [mm/mm]	σ_true max_ [MPa]	ε_true max_ [mm/mm]	E [MPa]
	b [mm]	t [mm]					
Ambient medium							
#1	6	3	0.8710	17.4687	16.0818	2.9161	0.0175
#2	6	3	0.9623	17.4687	17.7717	2.9161	0.0193
#3	6	3	0.9167	17.4690	16.9305	2.9161	0.0172
#4	6	3	0.9504	17.4689	17.5248	2.9161	0.0195
#5	6	3	0.9302	17.4689	17.1760	2.9161	0.0197
*Average value*			0.9261	17.4689	17.0970	2.9161	0.0187
*Standard mean square deviation*			0.0355	0.0002	0.6524	0.0000	0.0012
*Standard variation*			0.0013	0.0000	0.4257	0.0000	0.0000
Distilled water							
#1	6	3	0.9896	17.4688	18.2760	2.9161	0.0209
#2	6	3	0.9534	17.4688	17.6061	2.9161	0.0205
#3	6	3	0.9846	17.4687	18.1841	2.9161	0.0197
#4	6	3	1.0023	17.4687	18.5111	2.9161	0.0183
#5	6	3	0.9747	17.4688	18.0006	2.9161	0.0193
*Average value*			0.9809	17.4687	18.1156	2.9161	0.0197
*Standard mean square deviation*			0.0183	0.0000	0.3390	0.0000	0.0010
*Standard variation*			0.0003	0.0000	0.1149	0.0000	0.0000
Hydraulic oil							
#1	6	3	0.9551	17.4643	17.4881	2.9158	0.0205
#2	6	3	0.9588	17.4690	17.7067	2.9161	0.0192
#3	6	3	0.9408	17.4688	17.3746	2.9161	0.0205
#4	6	3	0.9812	17.4687	18.1206	2.9161	0.0192
#5	6	3	0.9726	17.4687	17.9628	2.9161	0.0196
*Average value*			0.9617	17.4679	17.7305	2.9160	0.0198
*Standard mean square deviation*			0.0157	0.0020	0.3131	0.0001	0.0006
*Standard variation*			0.0002	0.0000	0.0980	0.0000	0.0000
Cooling oil							
#1	6	3	0.8369	17.4688	15.4551	2.9161	0.0181
#2	6	3	0.8357	17.4689	15.4346	2.9161	0.0188
#3	6	3	0.8453	17.4689	15.6119	2.9161	0.0157
#4	6	3	0.8342	17.4688	15.4069	2.9161	0.0170
#5	6	3	0.8405	17.4688	15.5236	2.9161	0.0178
*Average value*			0.8386	17.4688	15.4864	2.9161	0.0175
*Standard mean square deviation*			0.0044	0.0001	0.0823	0.0000	0.0012
*Standard variation*			0.0000	0.0000	0.0068	0.0000	0.0000
UV							
#1	6	3	0.4470	10.4683	5.0419	2.4396	0.0616
#2	6	3	0.5218	12.6243	6.9511	2.6119	0.0428
#3	6	3	0.5356	11.3223	6.5432	2.5114	0.0447
#4	6	3	0.4681	11.0103	5.5271	2.4858	0.0358
#5	6	3	0.5919	12.4415	7.6906	2.5983	0.0259
*Average value*			0.5129	11.5733	6.3508	2.5294	0.0422
*Standard mean square deviation*			0.0574	0.9300	1.0705	0.0739	0.0132
*Standard variation*			0.0033	0.8648	1.1460	0.0055	0.0002

**Table 6:** Distilled water absorption of Ecoflex 00-10 test specimens.

No. Specimen	Mass after Drying [g]	Mass after 24 h in Distilled Water [g]	Absorption [%]
#1	5.94	6.12	3.03
#2	6	6.27	4.50
#3	6.08	6.25	2.80
#4	6.07	6.21	2.31
#5	6.17	6.25	1.30
Average value			2.79

**Table 7:** Hydraulic oil absorption of Ecoflex 00-10 test specimens.

No. Specimen	Mass after Drying [g]	Mass after 24 h in Distilled Water [g]	Absorption [%]
#1	6.12	6.68	9.15
#2	6.08	6.37	4.77
#3	5.84	6.25	7.02
#4	6.00	6.49	8.17
#5	6.28	6.44	2.55
Average value			6.33

**Table 8:** Cooling oil absorption of Ecoflex 00-10 test specimens.

No. Specimen	Mass after Drying [g]	Mass after 24 h in Distilled Water [g]	Absorption [%]
#1	5.8	6.41	10.52
#2	5.74	6.38	11.15
#3	5.54	6.54	17.63
#4	5.59	6.62	18.43
#5	5.74	6.34	10.45
Average value			13.65

## Data Availability

The data sets used in this study are available on request from corresponding.

## Appendix A

**Table A1:** Material characteristics of Ecoflex 00-10 and 00-30.

Characteristics	Ecoflex 00-10	Ecoflex 00-30
Shore hardness	00-10	00-30
Color	Translucent	Translucent
Manufacturer	Smooth-On	Smooth-On
Pot life	30 min	45 min
Cure time	4 h	4 h
Mix Ratio by Weight	1A:1B	1A:1B
